# Cost-Conscious of Anesthesia Physicians: An awareness survey

**DOI:** 10.12669/pjms.315.7520

**Published:** 2015

**Authors:** Sedat Hakimoglu, Volkan Hancı, Murat Karcıoglu, Kasım Tuzcu, Isıl Davarcı, Hasan Ali Kiraz, Selim Turhanoglu

**Affiliations:** 1 Sedat Hakimoğlu, MD, Assistant Professor, Department of Anesthesiology and Reanimation, Mustafa Kemal University, Faculty of Medicine, 31100, Serinyol, Antakya, Hatay, Turkey; 2 Volkan Hancı, MD, Associate Professor, Dept. of Anesthesiology and Reanimation, Dokuz Eylül University, Faculty of Medicine, İzmir, Turkey; 3 Murat Karcıoğlu, MD, Associate Professor, Department of Anesthesiology and Reanimation, Mustafa Kemal University, Faculty of Medicine, 31100, Serinyol, Antakya, Hatay, Turkey; 4 Kasım Tuzcu, MD, AssistantProfessor, Department of Anesthesiology and Reanimation, Mustafa Kemal University, Faculty of Medicine, 31100, Serinyol, Antakya, Hatay, Turkey; 5 Isıl Davarcı, MD, Assistant Professor, Department of Anesthesiology and Reanimation, Mustafa Kemal University, Faculty of Medicine, 31100, Serinyol, Antakya, Hatay, Turkey; 6 Hasan Ali Kiraz, MD, Assistant Professor, Dept. of Anesthesiology and Reanimation, ÇanakkaleOnsekiz Mart University, Faculty of Medicine, Çanakkale, Turkey; 7 Selim Turhanoğlu, MD, Professor, Department of Anesthesiology and Reanimation, Mustafa Kemal University, Faculty of Medicine, 31100, Serinyol, Antakya, Hatay, Turkey

**Keywords:** Anesthesia, Awareness, Cost effectively

## Abstract

**Objective:**

Increasing competitive pressure and health performance system in the hospitals result in pressure to reduce the resources allocated. The aim of this study was to evaluate the anesthesiology and intensive care physicians awareness of the cost of the materials used and to determine the factors that influence it.

**Methods:**

This survey was conducted between September 2012 and September 2013 after the approval of the local ethics committee. Overall 149 anesthetists were included in the study. Participants were asked to estimate the cost of 30 products used by anesthesiology and intensive care units.

**Results:**

One hundred forty nine doctors, 45% female and 55% male, participated in this study. Of the total 30 questions the averages of cost estimations were 5.8% accurate estimation, 35.13% underestimation and 59.16% overestimation. When the participants were divided into the different groups of institution, duration of working in this profession and sex, there were no statistically significant differences regarding accurate estimation. However, there was statistically significant difference in underestimation. In underestimation, there was no significant difference between 16-20 year group and >20 year group but these two groups have more price overestimation than the other groups (p=0.031). Furthermore, when all the participants were evaluated there were no significant difference between age-accurate cost estimation and profession time-accurate cost estimation.

**Conclusion:**

Anesthesiology and intensive care physicians in this survey have an insufficient awareness of the cost of the drugs and materials that they use. The institution and experience are not effective factors for accurate estimate. Programs for improving the health workers knowledge creating awareness of cost should be planned in order to use the resources more efficiently and cost effectively,

## INTRODUCTION

Current changes such as increasing pressure of competition, performance of the healthcare systems, and privatization of the healthcare sector are increasing expenses and reducing sources used for healthcare services. Performance coefficients and the expenses of healthcare service departments are not only taken into consideration as additional payments for healthcare providers by healthcare facilities, but their management is also increasingly important for healthcare facilities to survive.^1^ The success of a commercial corporation depends on its “ability to increase production when compared to the past by using available sources; in other words, its ability to increase productivity”. In this sense, behind the developed economies are conscious consumers. The drug and healthcare expenses of countries are closely correlated to their rate of economic growth.

In the current economic environment, healthcare systems and healthcare providers are under pressure to minimize their costs without compromising patient safety. Similar to other healthcare providers, anesthesiologists must deal with changes in third party payers and cost-containment pressures. Awareness of the prices of drugs and materials used is one of the primary factors that determine the cost.^2^

The awareness and knowledge of an anesthesiologist regarding the drugs and materials that they use is rather limited. To our knowledge, there is no study about this topic in our country so far. The aim of our study is to investigate the awareness of anesthesiologists and reanimation specialists employed in our country regarding the costs of materials.

## METHODS

This survey was conducted between September 2012 and September 2013 after approval by the Ethics Committee of the Mustafa Kemal University Medicine School (Head of the Ethics Committee: Selim Turhanoğlu; Approval date: 28.08.2012; Ethics Committee #:2012/07). The questionnaires were completed by anesthesiologists either via a face-to-face interview or using a web-based questionnaire form. The results of the web-based questionnaires from responders and those completed through face-to-face interviews were then assessed.

The inclusion criteria was an anesthesiology or reanimation specialist in Turkey. Other Physicians were excluded. The anonymity of participants and data confidentiality was protected.

**Fig.1:**
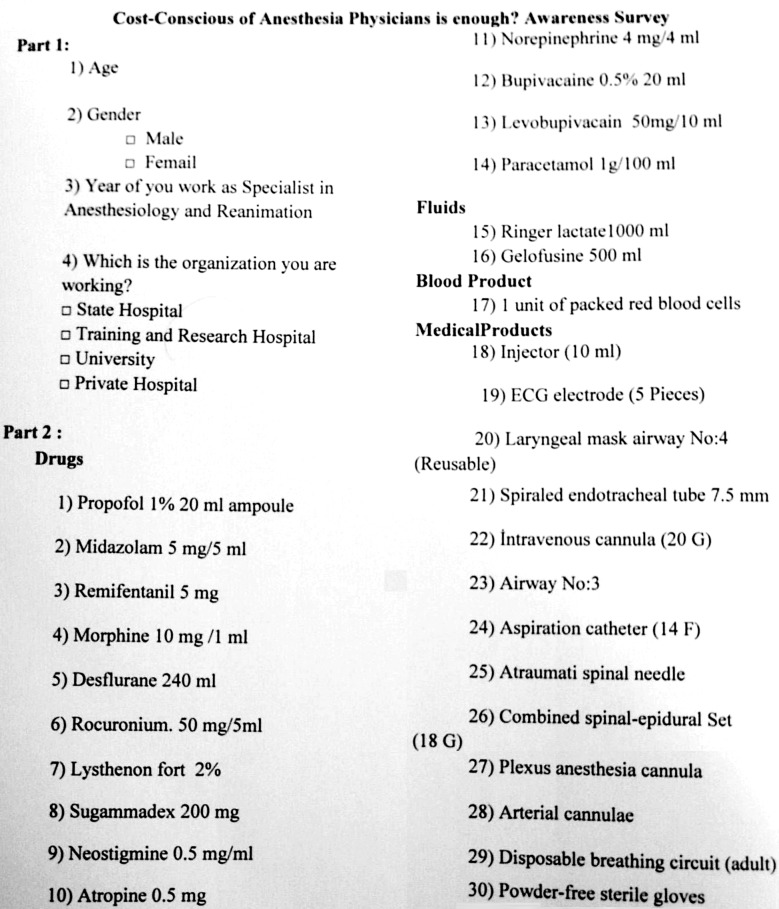
The detail of the components of the questionnaire.

The questionnaire consisted of two parts, including 30 items overall (Fig.1). The first part included informations regarding age, gender, facility, and length of employment as a specialist, while the second part included informations regarding drugs, intravenous fluids, blood product and medical products used in anesthesia practice. The actual prices of these items were determined by evaluating commercial invoices and interviewing manufacturers and hospital pharmacies. Estimates within the range of ±5% of the actual prices were considered as accurate estimates. Estimates 5% higher than the actual prices were considered as overestimations, while those 5% lower than the actual prices were considered as underestimations (Table-I).

**Table-I:** The list of products in the survey and their accurate price range (±5%).

		Accurate Price (±5% €)
Drugs	Propofol 1% 20 ml	0.49-0.54
	Midazolam 5 mg/5 ml	0.61-0.69
	Remifentanil 5 mg	9.82-10.85
	Morphine 10 mg 1 ml	0.13-0.15
	Desflurane 240 ml	29.12-32.17
	Rocuronium. 50 mg/5ml	1.59-1.75
	Lysthenonfort 2%	0.22-0.25
	Sugammadex200 mg	45.7-50.51
	Neostigmine0.5 mg/ml	0.11-0.12
	Atropine 0.5 mg	0.09-0.1
	Norepinephrine 4 mg/4 ml	2.44-2.7
	Bupivacaine 0.5% 20 ml	1.48-1.64
	Levobupivacain 50mg/10 ml	1.35-1.49
	Paracetamol 1g/100 ml	1.54-1.49
Fluids	Ringer Lactate1000 ml	1.55-1.71
	Gelofusine 500 ml	2.68-2.96
Blood Product	1 unit of packed red blood cells	29.84-32,98
Medical Products	Injector10 ml	0.04-0.047
	ECG electrode (5 Pieces)	0.33-0.37
	Laryngeal mask airway No: 4 (Reusable)	27.93-30.87
	Endotracheal Tube (Spiraled)7.5 mm	3.55-3.89
	İntravenous Cannula (Pink) 20 G	0.9-0.1
	Airway No: 3 (Green)	0.1-0.11
	Aspiration Catheter 14 F (Green)	0.06-0.07
	Spinal Needle (Tippedatraumatic)	4.42-4.88
	Combined Spinal-Epidural Set 18 G	12.07-13.51
	Plexus Anesthesia Cannula	12.07-13.51
	Arterial Cannulae	5.54-6.12
	Disposable Breathing Circuit (Adult)	15.94-17.62
	Sterile Gloves (Powder-free)	0.51-0.57

### Statistical Analysis

Data were analyzed by using SPSS for Windows version 15.0. Distribution of the data was assessed by using the Kolmogorov-Smirnov test. Independent groups with abnormal distribution were compared by using the Kruskal Wallis test, while paired comparisons within groups were performed using the Mann Whitney U test. Data with normal distribution were compared by using the student t-test. Correlations including age-accurate estimations or length of employment-accurate estimations were analyzed by using Spearman’s correlation coefficient. p<0.05 was considered as significant in all tests.

## RESULTS

Overall, 149 anesthesiology and reanimation specialists participated in the study, including 59 physicians (39.59%) from academic centers, 20 physicians (13.42%) from teaching hospitals, 33 physicians (22.14%) from state hospitals, and 37 physicians (24.83%) from private centers. There were 67 female (45%) and 82 male (55%) participants, with a mean age of 39 years. In 149 cases, the mean number of accurate estimations was 2 (5.8%; range: 0-6), while the mean numbers of underestimation and overestimation were 10 (35.13%; range: 1-27) and 18 (59.06; range: 2-28), respectively (Table-II).

**Table-II:** The distribution of demographic data and answers of the respondents.

N	149	
Age [Median (Min-Max)]	39 (27-63)	
Professional Year [Median (Min-Max)]	8 (1-32)	
Male [N (%)]	82	55
Female [N (%)]	67	45
Accurate Priced [Median (Min-Max)+(%)]	2 (0-6)	5.8
Low Priced [Median (Min-Max)+(%)]	10 (1-27)	35.13
Over Priced [Median (Min-Max)+(%)]	18 (2-28)	59.06

**Fig.2:**
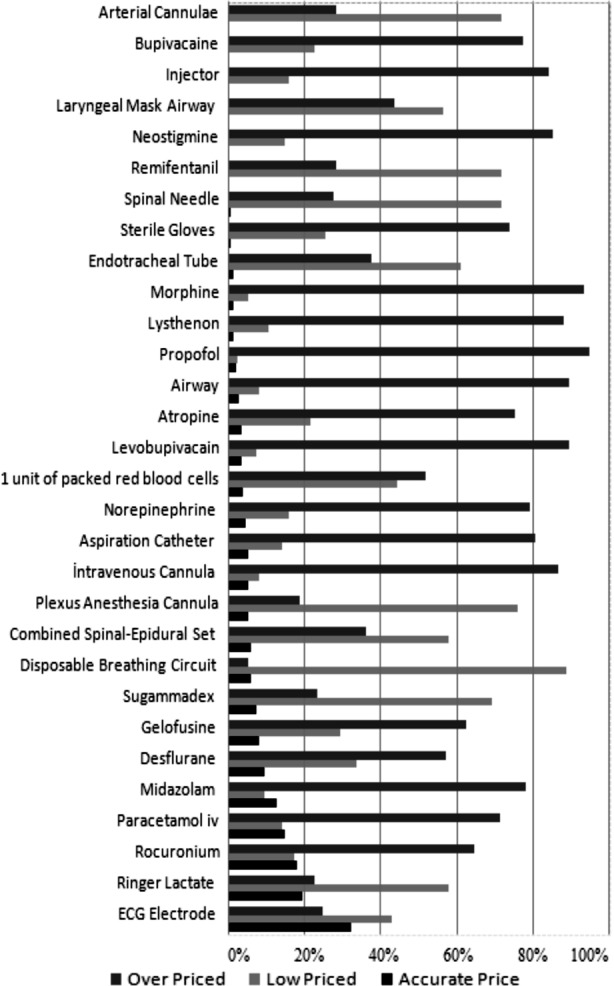
Distribution of answers to survey questions.

According to the responses of the anesthesiologists, the prices of the drugs and other materials (e.g., respiratory circuit, plexus cannula set, atraumatic spinal needle, combined spino-epidural set, laryngeal mask, arterial cannula, remifentanil, and Sugammadex) considered as expensive were underestimated compared to their actual prices (Fig.2).

When the participants were stratified according to the healthcare facility where they work, there was no significant difference among groups in regards to accurate estimation (p=0.883), underestimation (p=0.569) or overestimation of the prices (p=0.621) (Table III and IV). It was found that the mean time in specialty (p=.032) and age (p=0.002) were different when compared among groups. The mean time in specialty was found to be different in participants working at private facilities (10 years; range: 3-32 years) when compared to those working at academic centers, teaching hospitals, and county hospitals (p=0007). In addition, there was a difference in age between clinicians working at private facilities (43 years; range: 29-63) and those working at a state hospital (37 years; range: 27-61 years) (p=0.01).

**Table-III:** The distribution of age and Professional year according the institution.

		Year	Age
Institution	N (%)	Median (Min-Max)	Median (Min-Max)
University	59 (39.59)	8 (1-32)	39 (30-62)
Training and Research Hospital	20 (13.42)	7.5 (1-30)	39 (28-58)
State Hospital	33 (22.14)	4 (1-31)	37 (27-61)
Private Hospital	37 (24.83)	10 (3-32)	43 (29-63)

**Table-IV:** Comparison of responses studied grouped according to the institution.

	Accurate Priced	Over Priced	Low Priced
Institution	Median (Min-Max)	Median (Min-Max)	Median (Min-Max)
University	2 (0-6)	18 (2-26)	11 (4-27)
Training and Research Hospital	1.5 (0-3)	18.5 (13-27)	10 (2-14)
State Hospital	1 (0-4)	19 (8-28)	10 (1-20)
Private Hospital	1 (0-5)	18 (10-27)	10 (2-20)
P	0.883	0.621	0.569

When the participants were stratified according to mean time in specialty, it was found that mean time in specialty was 0-5 years in 35.57%, 6-10 years in 30.20%, 11-15 years in 16.10%, 16-20 years in 8.72%, and over 20 years in 9.39% of the participants. There was no significant difference among these groups regarding estimation of accurate price (p=0.564) and overestimation of price (p=0.091) (Table-V). However, there was a significant difference as regards to underestimation of price (p=0.031). There was no significant difference between those with experience of 16-20 years and those with over 20 years of experience regarding the underestimation of prices, while the number of underestimations was higher in other groups. When the anesthesiologists were compared according to gender, there was no significant difference between female and male clinicians regarding the estimation of accurate price, or in regards to the overestimation and underestimation of price. No significant correlation was detected using Spearman’s analysis regarding the age-estimation of accurate price and the mean time in specialty-estimation of accurate price [r=0.35 and r=0.132, respectively].

**Table-V:** Comparison of responses grouped according to their Professional year (* P<0.05).

		Accurate Priced	Over Priced	Low Priced
Professional Year	N (%)	Median (Min-Max)	Median (Min-Max)	Median (Min-Max)
0-5	53 (35.57)	1 (0-6)	19 (8-28)	9 (1-20)
6-10	45 (30.20)	2 (0-5)	19 (9-27)	10 (2-20)
11-15	24 (16.10)	2 (0-6)	18.5 (11-26)	9.5 (3-14)
16-20	13 (8.72)	1 (0-3)	13 (2-25)	14 (5-27)*
>20	14 (9.39)	1.5 (0-5)	18.5 (2-18)	9.5 (2-18)
P		0.564	0.091	0.031

**Table-VI:** Comparison of responses grouped according to gender.

	Accurate Priced	Over Priced	Low Priced
Gender (N)	Mean±SD	Mean±SD	Mean±SD
Female (N=67)	1.87±1.32	19.13±4.08	9±3.75
Male (N=82)	1.63±1.18	16.56±4.8	11.79±4.64
P	0.384	0.098	0.066

## DISCUSSION

In investigating the awareness of anesthesiology and reanimation specialists employed in our country regarding the prices of materials they use and the factors influencing their awareness of such, the mean percentage of accurate price estimation was 5.8% among all 30 items, while the mean percentages of underestimation and overestimation of price were 35.13% and 59.06%, respectively. When participants were stratified according to gender, the facility in which they work, and their mean time in specialty, there was no significant difference in the percentage of accurate price estimations among the groups. No significant difference was found between 16-20 years and >20 years of experience in a specialty in regards to underestimation, and the number of underestimations were higher compared to other groups (p=0.031). Moreover, there was no significant correlation between age and accurate price estimation or between the mean time in specialty and accurate price estimation when all participants were included in the analysis.

Healthcare expenses today comprise a substantial part of the national expenditure.^3^ Similar to other sectors, the uncontrolled use of material and drugs in the healthcare field leads to an increase in healthcare expenses. Administrators of clinical anesthesia have shown that, among all expenses, the highest price increases have occurred in anesthesia agents and equipment.^4^ Particularly, US market research studies show that the sale of anesthetic agents has increased by 9.2% per year.^4^ The drug charges of the Turkish Social Security Institution have increased from 4.3 billion lira in 2002, to 8.85 billion lira in 2007, to 12 billion lira in 2008.^3^ Awareness of items prices will change the total cost of treatment. Thus, anesthesiologists should have greater knowledge about the prices of the materials that they use. Inadequate awareness of the prices of drugs and materials used can lead to procedures that are not cost–effective. In the present study, we focused on elements (drugs, fluids, blood product and medical products) that are considered to be important in regards to the overall costs of anesthesia procedures and affecting factors.

In previous studies it has been reported that there is inadequate awareness as regards to such costs.^5^-^9^ In a study using a 25-item questionnaire about drugs used in anesthesia practice, Javasuriya et al. showed that there was inadequate awareness regarding costs and that this was not affected by experience.^9^ The authors also noted that all estimations of prices were below the actual prices.^9^ In a survey of 50 anesthesia providers, Hadjipavlou et al.^8^ reported that expensive drugs were priced as inexpensive, while inexpensive ones were priced as expensive. In addition, the authors showed that time in a given occupation had no correlation with an accurate estimation. In our study, the percentage of underestimation was high for expensive products (e.g., respiratory circuit, plexus cannula set, atraumatic spinal needle, combined spino-epidural set, laryngeal mask, arterial cannula, remifentanil, and Sugammadex). The number of accurate estimations was rather low (5.8%) in our study. In addition, accurate estimation was not affected by the mean time in specialty. The percentage of overestimation was 59.06%. Perception of the materials as more expensive than their actual prices corresponded to efforts to minimize expenses due to performance concerns.

In our study, the percentage of underestimation was 35.13%. Lethbridge and Secker Walker have reported that the continuous underestimation of prices is the main reason for increasing expenses.^10^ Although remarkable errors were observed in the estimation of costs, the number of overestimations was higher than underestimations among the anesthesiology and reanimation specialists in our country. There was a higher number of underestimations among clinicians with 16-20 years of experience compared to other groups which can be explained by the fact that anesthesia and reanimation specialists with 16-20 years of experience are a high-risk group for exhaustion. The incidence of burnout in anesthesiology and reanimation specialists who have more than 16 years experience is high because of lack of satisfaction in working conditions, long working hours and lack of sleep^11^. The most commonly cited reasons for inaccurate estimations include inadequacy in terms of cost consciousness and economic education.^12^,^13^ It has been reported that, in addition to education programs, developing practical guidelines remarkably decreases expenses without influencing clinical outcomes.^14^

In contrast to other studies, no significant difference was found in the numbers of accurate estimations, overestimations or underestimations when the anesthesiology and reanimation specialists were stratified according to their facility and gender. The lack of difference among the groups stratified according to facility indicates that education required is not provided at facilities.

Competition amongst healthcare facilities depends on their “ability to increase production when compared to the past by using available sources; in other words, their ability to increase productivity”. In fact, behind the developed economies can be said that there are conscious consumers. The selection of procedures with mild to moderate costs by anesthesiologists, in addition to the implementation of appropriate educational programs, is essential to reduce expenses.^2^,^15^ Healthcare expenses today comprise a substantial part of the national expenditure.^3^ The uncontrolled use of materials and drugs by healthcare providers contributes to the increase in healthcare expenses. Similar to other healthcare providers, anesthesia and reanimation specialists should be conscious to what degree their drug and material use, as well as their expected costs can be reduced. With developments in medicine and medical technologies, hospitals have changed rapidly to have increasing importance as socioeconomic institutions that consume a significant amount of resources.

In conclusion, awareness as to the prices of the drugs and materials used in clinics was inadequate among anesthesiology and reanimation specialists who responded to the questionnaire of this study. Similar to other healthcare providers, anesthesia and reanimation specialists should be conscious as to what degree their drug and material use, as well as their expected costs can be reduced. One’s work facility and length of employment are not important factors for the accurate estimation of prices. For the effective and efficient use of institutional resources by healthcare providers, training programs and guidelines should be implemented to develop cost consciousness.

## References

[1] Bauer M, Martin E (1999). Management undorganisationsentwicklungim Krankenhaus. Anaesthesist.

[2] Valenzuela RC, Johnstone RE (1997). Cost containment in anesthesiology: a survey of department activities. J ClinAnesth.

[3] Pınar N (2012). Drug expenditures in our country. J Turgut Ozal Med Center.

[4] Johnstone RE, Jozefczyk KG (1994). Costs of anesthetic drugs: experiences with a cost education trial. Anesth Analg.

[5] Simpson M (1978). The cost of anaesthetic drugs and equipment Anaesthesia.

[6] Bailey CR, Ruggier R, Cashman JN (1993). Anaesthesia: cheap at twice the price? Staff awareness, cost comparisons and recommendations for economic savings. Anaesthesia.

[7] Simonsen MS, Spangsberg NLM, Carlsson P (1999). Cost consciousness among anesthetic staff Acta Anaesthesiol Scand.

[8] Hadjipavlou M, Bailey CR (2010). The price of everything and the value of nothing: cost awareness in anaesthesia. J PerioperPract.

[9] Jayasuriya JP (1990). Cost awareness among junior anaesthetists in Sri Lanka. Ceylon Med J.

[10] Lethbridge JR, Secker Walker J (1986). Cost of anaesthetic drugs and clinical budgeting. BrMed J.

[11] Beyhan S, Gunes Y, Türktan M, Ozcengiz D (2013). Investigation of the Burn out Syndrome Among the Eastern Mediterranean Region Anaesthesiologists. Turk J Anaesth Reanim.

[12] Thomas DR, Davis KM (1987). Physici anawareness of costunder prospective reimbursement systems. Med Care.

[13] Wickings I, Coles JM, Flux R, Howard L (1983). Review of clinical budgeting and costing experiments. Br Med J.

[14] Lubarsky DA, Glass PSA, Ginsberg B, Dear GL, Dentz ME, Gan TJ (1997). The successful implementation of pharmaceutical practice guidelines. Anesthesiology.

[15] Mills G, Chaffe A (1993). Is cost-awareness really improving?. Health Trends.

